# Functional Status and Health-Related Quality of Life in Patients with Peripheral Artery Disease: A Cross-Sectional Study

**DOI:** 10.3390/ijerph182010941

**Published:** 2021-10-18

**Authors:** Mihui Kim, Yesol Kim, Gi Wook Ryu, Mona Choi

**Affiliations:** 1 College of Nursing and Brain Korea 21 FOUR Project, Yonsei University, Seoul 03772, Korea; ystelra50@gmail.com (M.K.); yesolkim@yuhs.ac (Y.K.); gw14@nsu.ac.kr (G.W.R.); 2 Department of Nursing, Namseoul University, Cheonan-si 31020, Korea; 3 College of Nursing and Mo-Im Kim Nursing Research Institute, Yonsei University, Seoul 03772, Korea

**Keywords:** peripheral artery disease, functional status, health-related quality of life, EQ-5D-5L

## Abstract

Peripheral artery disease (PAD) is a progressive atherosclerotic disease that negatively affects individuals’ functional status and health-related quality of life (HRQOL). This study aimed to investigate the HRQOL and associated factors in patients with PAD in Korea. We conducted a cross-sectional study using self-administered questionnaires in a tertiary hospital in Seoul. We measured HRQOL using the EuroQol-5 dimensions-5 levels (EQ-5D-5L) questionnaire and the functional status using a PAD-specific walking impairment questionnaire. We also measured health behavior, social support, walking impairment, general health perceptions, and clinical characteristics; lastly, we performed a descriptive analysis and multiple linear regression analysis. Participants of this study comprised 138 patients (mean age 69.04 ± 10.94 years; men 91.3%). The mean EQ-5D-5L utility score was 0.81 ± 0.17. The patients’ HRQOL was significantly associated with claudication pain (β = −0.188, *p* = 0.012), walking distance (β = 0.371, *p* < 0.001), and stair climbing (β = 0.315, *p* = 0.001). The regression model predicted 60.4% of patients’ HRQOL (F = 15.92, *p* < 0.001). Our study showed that less severe claudication pain and a low degree of difficulty in walking distance and stair climbing were significantly related to better HRQOL. To enhance patients’ HRQOL, health professionals should focus on managing symptoms and improving functional status.

## 1. Introduction

Peripheral artery disease (PAD) is a progressive atherosclerotic disease that causes stenosis or obstruction of the peripheral arteries [1]. Globally, an estimated 236.62 million people older than 25 years live with PAD [2], exhibiting either no symptoms or mild to severe symptoms [3]; the prevalence increases substantially with age [1]. Approximately two-thirds of patients with PAD are asymptomatic [4]. A classic symptom of PAD is claudication, which is characterized by fatigue, discomfort, cramps, pain, or numbness in the calves, thighs, or buttocks when walking and is consistently relieved by rest [5,6]. Other severe symptoms include ischemic rest pain, ulceration, and gangrene in one or both legs, which may lead to amputation if left untreated [5].

Functional status is patients’ capacity to take part in community-based, daily activities, including walking ability [7]. Functional impairment related to walking ability is present even in patients with asymptomatic PAD [4,8]. This impairment negatively impacts mobility, social participation, health-related quality of life (HRQOL), and mortality [6,9,10]. Therefore, the treatment goals for PAD include managing its symptoms, alleviating functional impairment to prevent the risk of cardiovascular and limb events, preserving functional status, and improving HRQOL [7,8,11]. In this sense, assessing patients’ symptoms, functional status, and HRQOL should be incorporated into evaluating all treatments.

Recent studies have increasingly emphasized measuring HRQOL derived directly from patients to represent health conditions comprehensively [7,9,12]. To date, HRQOL-related studies of PAD mainly evaluate the effectiveness of treatment [12,13,14] or focus on symptomatic PAD [15,16]. A previous study indicated that there is no comprehensive study of associated factors affecting HRQOL in patients with PAD, regardless of symptoms. It is essential to identify associated factors to develop nursing interventions aimed at improving HRQOL in patients with PAD. Accordingly, this study aimed to assess the level of HRQOL and investigate the associated factors among patients with PAD.

To guide this study, we used the revised Wilson and Cleary’s HRQOL model by Ferrans and colleagues [17,18] to measure HRQOL and identify its predictors in patients with PAD. This model provides a comprehensive picture of the nature and understanding of the structural causal associations between factors [17,18]. Additionally, it was found that personal values and preferences expressed as life satisfaction influence the overall quality of life [18]. This model focused primarily on five components of patient outcomes: biological function, symptoms, functional status, general health perceptions, and overall quality of life at both individual and environmental levels [18]. We assessed and analyzed all the multidimensional variables in this model.

## 2. Materials and Methods

### 2.1. Study Design and Participants

We conducted a descriptive, cross-sectional study. We used convenience sampling to recruit patients from the cardiovascular outpatient clinic of a tertiary hospital in Seoul, Korea. This study included 138 patients with PAD, recruited from June to October 2020. The sample size was calculated based on an effect size of 0.15, a significance level of 0.05, power of 0.8, and 13 predictors for multiple regression using G *Power software 3.1.9.2 [19]. A total of 131 participants were estimated, and the final sample size was 155, considering a dropout of 15%. Inclusion criteria were those who (a) were older than 19 years, (b) were diagnosed with PAD by a cardiologist, and (c) visited the outpatient clinic for treatment or follow-up. Exclusion criteria were those who (a) underwent major leg amputation, (b) were unable to walk independently, (c) had a history of chronic cancer, or (d) had a severe psychiatric disease. The outpatient nurse informed researchers of possible participants when patients who met the eligible criteria visited the clinic. Researchers explained this study’s purpose and process in a separate room and obtained written informed consent from patients who voluntarily agreed to participate in this study. Patients completed a structured, self-reporting questionnaire.

### 2.2. Measurements

#### 2.2.1. Individual Characteristics

We acquired data regarding participants’ characteristics, including age, sex, body mass index (BMI), marital status, employment status, education level, alcohol consumption, and smoking. We also measured health behavior in five dimensions using the Cardiac Health Behavior Scale for Korean adults (CHB-K21) [20]. The scale comprises 21 items, with responses ranging from 1–4 points on a Likert scale. A higher score indicates more favorable health behaviors. The scale consists of five items for health responsibility, four items for physical activity, six items for dietary habits (eating habit and food choice), three items for stress management, and three items for smoking cessation. The Cronbach’s α of the Korean version was 0.83 [20] and 0.81 in this study.

#### 2.2.2. Biological Function

To measure patients’ biological function, we used clinical characteristics, such as the duration of PAD, comorbidities, experienced symptoms, family history of PAD and cardiovascular disease, the use of a walking assistive device, and PAD treatment history.

#### 2.2.3. Functional Status and Symptoms

Functional status was assessed using the Walking Impairment Questionnaire (WIQ) [21], a widely used PAD-specific questionnaire [9], to measure patients’ perceptions of their difficulty in walking. The WIQ measures walking ability, including distance, speed, and stair climbing, and further evaluates symptoms that indicate walking difficulty due to claudication. We used the Korean version of the WIQ [22], which consists of walking distance (seven items), walking speed (four items), stair climbing (three items), and pain (two items). Responses are rated on a five-point Likert scale ranging from 0 (great difficulty or unable to do) to 4 (no difficulty). Lower scores indicate that patients perceived a high degree of difficulty when walking. The symptom score was reverse coded so that higher scores presented a perceived high degree of claudication. Each item’s weighting formulas determined functional status, including walking distance, walking speed, and stair climbing scores [22].

#### 2.2.4. General Health Perceptions

We measured perceived health status and perceived PAD severity to assess general health perceptions. Perceived health status included one item that assesses patients’ perceived health status, with responses rated on a five-point Likert scale from 1 (very bad) to 5 (very good). A higher score indicates a more positive health perception. Perceived PAD severity was measured by one item regarding how patients think about their disease severity, rated on a five-point Likert scale from 1 (not at all) to 5 (very severe). A higher score indicates that the patient perceived the disease as more serious.

#### 2.2.5. Health-Related Quality of Life

We measured HRQOL using the EuroQol-5 dimensions-5 levels (EQ-5D-5L) questionnaire [23], which consists of two parts: the EQ-5D descriptive system and EQ visual analog scale (VAS). The EQ-5D-5L, developed by the EuroQol group [23], is a comprehensive instrument for describing and valuing health [24] and measuring patients’ preferences related to health [25]. The EQ-5D questionnaire consists of five dimensions: mobility, self-care, usual activities, pain/discomfort, and anxiety/depression. Each item is rated on five levels: no problems, slight problems, moderate problems, severe problems, and extreme problems. EQ-VAS measures patients’ perception of health using a vertical visual analog scale from 0 to 100. A higher score reflects a more favorable perception of their health status. The Korean value set that weighted the EQ-5D index values used the composite time trade-off [25]. A utility value of 0 represents a health state equivalent to death, and a value of 1 represents perfect health [23]. The possible range of utility values in the Korean version is −0.066 to 1.00 [25]. We obtained permission from the EuroQol group to use the Korean version.

#### 2.2.6. Environmental Characteristics

We measured perceived social support offered by a special person (spouse or partner), family members, and friends using the 12-item Multidimensional Scale of Perceived Social Support (MSPSS) [26]. Responses are rated on a seven-point Likert scale ranging from 1 (very strongly disagree) to 7 (very strongly agree). We summed the scores of all items and then divided that value by the number of items to calculate the mean score. Total scores can range from 1–7, with higher scores representing higher levels of perceived social support. The reliability and validity of the Korean version were verified [27]. The Cronbach’s α was 0.88 at the time of development [26], 0.90 of the Korean version [27], and 0.86 in this study.

### 2.3. Statistical Analysis

This study used descriptive statistics to analyze individual characteristics, biological function, symptoms, functional status, general health perceptions, and HRQOL using frequencies, percentages, mean, and standard deviation (SD). We performed a *t*-test and analyses of variance (ANOVA) to analyze differences in HRQOL according to individual characteristics. We then analyzed relationships among HRQOL and associated factors using Pearson’s correlation coefficient. Finally, we performed a multiple linear regression analysis with an Enter method to identify the factors associated with patients’ HRQOL. All significant variables based on *t*-test, ANOVA, and Pearson’s correlation were entered into the multiple linear regression model to control for confounding variables. For all tests, we considered a *p*-value < 0.05 to be statistically significant. Statistical analyses were performed using IBM SPSS Statistics for Window, Version 25.0 (Armonk, NY, USA).

### 2.4. Ethical Consideration

This study was approved by the Institutional Review Board (IRB) of the Yonsei University Health System (IRB number: 4-2020-0299). All participants signed an informed consent form prior to participating in the study. The investigation conforms with the principles outlined in the Declaration of Helsinki.

## 3. Results

### 3.1. Study Design and Participants

Of the 150 patients with PAD who completed our questionnaires, we excluded 12 patients from the analysis owing to missing data; thus, 138 patients participated in the study. Table 1 presents the demographic and clinical characteristics of the patients with PAD.

The mean age of patients was 69.04 (±10.94) years, and 91.3% were men. More than 50% were overweight or obese. Almost half of the patients were unemployed. A total of 49 patients (35.5%) were high school graduates. There were 66 patients (47.8%) who were current drinkers, and 32 patients (23.2%) were current smokers. The mean duration of PAD was 8.36 years (IQR: 2.75–11.00). Considering comorbidities, patients diagnosed with diabetes and hypertension were 70 (50.7%) and 92 (66.7%), respectively. During treatment, most patients experienced intermittent claudication, and 40 patients (29.0%) experienced critical limb ischemia, such as rest pain, delayed wound healing, and ulcers or gangrene. Fifteen patients (10.9%) used a walking assistive device. Approximately 85% of patients underwent endovascular therapy, 23 patients (16.7%) received surgical bypass, and 9 patients (6.5%) experienced minor amputation.

To examine the association of demographic and clinical characteristics and HRQOL, we compared the EQ-5D-5L scores among patients. The EQ-5D-5L utility mean scores of all patients were 0.81 (± 0.17), and there were statistically significant differences in education level and using a walking assistive device. The EQ-5D-5L utility mean scores for patients with education lower than high school graduation was 0.75, lower than other groups (F = 3.713, *p* = 0.027). The utility mean scores of EQ-5D-5L in the walking assistive device group was 0.64, lower than for those not using any walking assistive device (*p* = 0.006).

### 3.2. Associated Factors for HRQOL

The mean scores for health behavior, social support, claudication pain, functional status, general health perception, and HRQOL are presented in Table 2.

The mean scores for health behavior and social support were 2.83 ± 0.46 and 5.42 ± 1.07, respectively. The mean score for claudication pain was 1.20 ± 1.22. A total of 63% of patients reported walking difficulty due to claudication pain. The speed score presented the lowest mean score of functional status (0.51 ± 0.28). The mean scores for perceived health status and PAD severity were 2.78 ± 0.79 and 3.65 ± 1.09, respectively. The mean score for EQ-5D-5L utility score was 0.81 ± 0.17 (range: 0.16–1.00), and VAS was 66.84 ± 18.99.

Among the five dimensions of HRQOL, the smallest number of patients who answered “no problem” reported issues with mobility and pain/discomfort. In addition, the dimension with the greatest number of patients who responded “no problem” was self-care (Table 3). Approximately 60% of patients reported problems with both mobility and pain/discomfort dimensions.

### 3.3. Correlation between Influencing Factors and HRQOL

**Figure 1:**
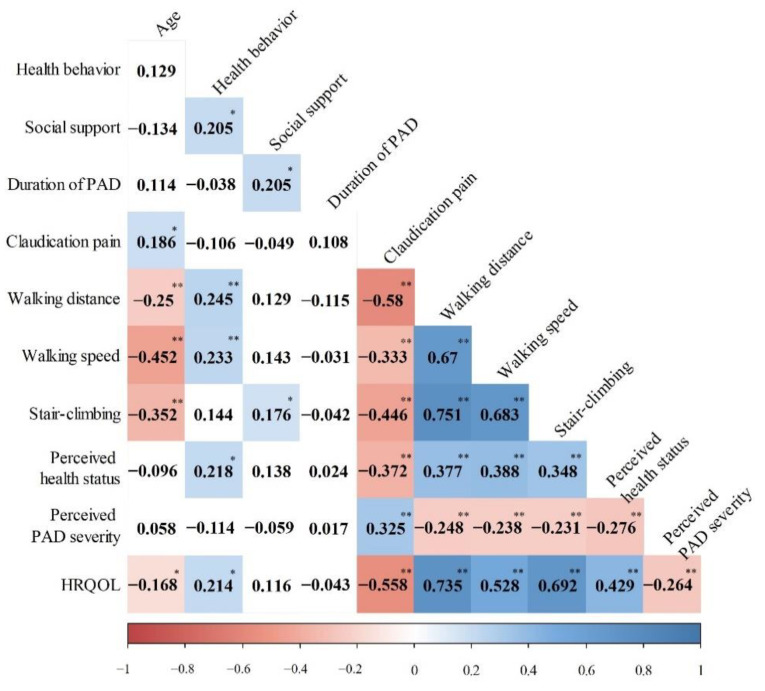
Correlation between influencing factors and HRQOL. Note: HRQOL, health-related quality of life; PAD, peripheral artery disease; * *p* < 0.05, ** *p* < 0.001.

Patients’ HRQOL was positively correlated with health behavior (r = 0.214, *p* = 0.012), walking distance (r = 0.735, *p* < 0.001), walking speed (r = 0.528, *p* < 0.001), stair climbing (r = 0.692, *p* < 0.001), and perceived health status (r = 0.429, *p* < 0.001). However, it was negatively correlated with age (r = −0.168, *p* = 0.049), claudication pain (r = −0.558, *p* < 0.001), and perceived PAD severity (r = −0.264, *p* = 0.002). Social support and duration of PAD showed no significant correlation with HRQOL (Figure 1). The patients’ EQ-5D-5L scores positively correlated with the EQ-VAS scores (r = 0.553, *p* < 0.001).

### 3.4. Multiple Linear Regression Analyses for HRQOL

We performed multiple linear regression to identify the factors associated with HRQOL in patients with PAD. The independent variables entered in the regression model included age, sex, education level, duration of PAD, use of walking assistive device, health behavior, claudication pain, functional status, general health perception, and social support. A variance inflation factor (VIF) was used to detect the degree of multicollinearity of the independent variables. The VIFs for all independent variables were below 10 (range: 1.126–3.361), indicating no multicollinearity problem. A higher HRQOL was significantly associated with less severe claudication pain (β = −0.188, *p* = 0.012) and a low degree of difficulty in walking distance (β = 0.371, *p* < 0.001) and stair climbing (β = 0.315, *p* = 0.001; Table 4). This regression model explained 60.4% of the variance in HRQOL of patients with PAD (F = 15.92, *p* < 0.001).

## 4. Discussion

This study aimed to assess the HRQOL of patients with PAD and carried out a comprehensive investigation to identify its associated factors based on Ferrans and colleagues’ HRQOL model. Our results showed that functional status and symptoms were associated factors of HRQOL in patients with PAD. We used the EQ-5D-5L to measure HRQOL in patients with PAD, a comprehensive instrument for describing and valuing health [24] and measuring patients’ preferences related to health [25]. In this study, the EQ-5D-5L utility score estimated HRQOL at a value of 0.81. This result is higher than the estimated EQ-5D utility scores of 0.64 for those diagnosed with PAD within one year [23,28], 0.73 for symptomatic patients with PAD [29], and 0.79 for chronic obstructive pulmonary disease in patients living with PAD [30]. However, this is lower than the estimated EQ-5D utility scores of 0.87 for the general population over 60 years in Korea [31]. This result suggests that PAD is a risk factor in lowering HRQOL and that HRQOL could be further reduced depending on comorbid diseases or symptoms.

The results of this study indicate that HRQOL is significantly correlated with age, health behavior, claudication pain, functional status (walking distance, walking speed, and stair climbing), perceived health status, and perceived PAD severity. The multiple linear regression analysis showed that claudication pain and functional status (walking distance and stair climbing) were consistently associated with HRQOL. In other words, less severe claudication pain and a low degree of difficulty in walking distance and stair climbing strongly predicted better HRQOL in patients with PAD (R^2^ = 0.604). These results differ from previous studies that report that depression, discomfort, and perceived health status were independent predictors of HRQOL in older adults with chronic diseases [32]. PAD is a chronic disease characterized by intermittent claudication and declined mobility. This result emphasizes assessing and managing the HRQOL of patients with PAD as an independent outcome of improved subjective symptoms and functional status in exercise interventions, endovascular therapy, and medical treatments.

In our sample, claudication pain tended to affect HRQOL, and 63.0% of our participants have walking difficulty due to claudication. Several studies have recommended supervised or structured physical exercise training, endovascular therapy, and medical treatment as the most effective strategies for improving limb symptoms [7,8]. Therefore, applying effective strategies—alone or in combination—after comparing the benefits and risks in terms of patients’ symptom degrees could be beneficial in managing the symptoms and improve their HRQOL.

Walking distance and stair climbing also affected HRQOL. The percentages of participants who showed difficulty in walking a distance of 200 m, walking at an average speed, and climbing three flights of stairs were 37.7%, 54.7%, and 59.4%, respectively. According to previous meta-reviews, structured exercise interventions improved objective walking performance and subjective functional status [13,33]. Therefore, to improve the HRQOL for PAD patients, healthcare providers should establish strategies to improve walking performance and subjective functional status through structured exercise interventions.

Our study has several limitations. First, using convenience sampling, we recruited participants diagnosed with PAD who were receiving treatment in a single tertiary hospital. This means that PAD was defined by patients’ medical history and the clinical judgment by medical staff; moreover, 91.3% of the participants in the study were male, which did not reflect the sex ratio in PAD incidence. Therefore, this study is limited in generalizability even though we investigated a comprehensive influence factor affecting HRQOL using Ferrans and colleagues’ causal model. Second, disease progression and treatment process can change HRQOL; as this was a cross-sectional study, we could not report on the changes in HRQOL as the disease progressed. Finally, as these were self-reported questionnaires, we did not measure objective indicators, such as the ankle-brachial index. However, with the emphasis on measuring subject indicators directly derived from patients, it is possible to investigate factors affecting HRQOL in patients living with PAD.

## 5. Conclusions

Our study showed that less severe claudication pain and a low degree of difficulty in walking distance and stair climbing were significantly associated with better HRQOL in patients living with PAD. In this sense, physical function and status predict HRQOL more reliably than PAD severity and comorbid conditions. Therefore, to improve the HRQOL of individuals living with PAD, health professionals should focus on finding ways to manage symptoms and improve functional status. The study results can contribute to the development of evidence-based interventions to improve HRQOL in patients with PAD.

## Figures and Tables

**Table 1:** Demographic and clinical characteristics (*n* = 138).

Variables	Categories	Mean ± SD or *n* (%)	EQ-5D-5L Utility Score	*t* or F (*p*)
Age, years	Total	69.04 ± 10.94	0.81 ± 0.17	
	≤59	25 (18.1)	0.86 ± 0.12	1.327 (0.268)
	60–69	38 (27.5)	0.79 ± 0.19	
	70–79	50 (36.2)	0.80 ± 0.17	
	≥80	25 (18.1)	0.78 ± 0.17	
Sex	Male	126 (91.3)	0.81 ± 0.17	0.412 (0.681)
	Female	12 (8.7)	0.79 ± 0.19	
BMI, kg/m^2^	Total	23.40 ± 3.11		
	Normal (<23.0)	64 (46.4)	0.80 ± 0.17	1.130 (0.326)
	Overweight (23.0–24.9)	37 (26.8)	0.78 ± 0.21	
	Obese (≥25.0)	37 (26.8)	0.84 ± 0.09	
Marital status	Married	116 (84.1)	0.81 ± 0.16	0.101 (0.460)
	Not married	22 (15.9)	0.80 ± 0.18	
Employment	Employed	58 (42.1)	0.84 ± 0.14	1.791 (0.076)
	Unemployed	80 (58.0)	0.79 ± 0.18	
Education	<High school	46 (33.3)	0.75 ± 0.22	3.713 (0.027)
	High school	49 (35.5)	0.83 ± 0.13	
	College or higher	43 (31.2)	0.84 ± 0.13	
Current drinker	Yes	66 (47.8)	0.83 ± 0.14	1.783 (0.077)
Current smoker	Yes	32 (23.2)	0.82 ± 0.13	0.085 (0.919)
Duration of PAD, years	Total	8.36 ± 8.22		
	<8	85 (61.6)	0.81 ± 0.18	0.493 (0.623)
	≥8	53 (38.4)	0.80 ± 0.14	
Family history	PAD	3 (2.2)	0.86 ± 0.12	0.550 (0.583)
	Cerebrovascular disease	29 (21.0)	0.85 ± 0.12	1.733 (0.085)
Comorbidity ^†^	Diabetes	70 (50.7)	0.79 ± 0.19	−1.176 (0.242)
	Hypertension	92 (66.7)	0.81 ± 0.17	0.075 (0.940)
	Chronic kidney disease	31 (22.5)	0.79 ± 0.21	−0.574 (0.570)
	Cardiovascular disease	52 (37.7)	0.83 ± 0.14	1.217 (0.226)
	Cerebrovascular disease	12 (8.7)	0.82 ± 0.19	0.201 (0.841)
PAD symptoms experienced during treatment ^†^	Intermittent claudication	130 (94.2)	0.80 ± 0.17	−1.603 (0.111)
	Rest pain	13 (9.4)	0.67 ± 0.26	−2.044 (0.062)
	Delayed wound healing	35 (25.4)	0.76 ± 0.20	−1.818 (0.075)
	Ulcers or gangrene	20 (14.5)	0.76 ± 0.21	−1.435 (0.153)
	No pain	6 (4.3)	0.92 ± 0.09	−1.751 (0.082)
Using walking assistive device	Yes	15 (10.9)	0.64 ± 0.22	−3.203 (0.006)
	No	123 (89.1)	0.82 ± 0.15	
Treatment history ^†^	Endovascular therapy	116 (84.1)	0.81 ± 0.17	0.253 (0.800)
	Surgical bypass	23 (16.7)	0.78 ± 0.18	−0.888 (0.376)
	Minor amputation	9 (6.5)	0.80 ± 0.19	−0.198 (0.843)

Note. BMI, body mass index; PAD, peripheral artery disease; SD, standard deviation; ^†^ Multiple choice is possible.

**Table 2:** Associated factors with HRQOL in the theoretical model (*n* = 138).

Variables	Subcategories	*n* (%)	Score		
			Mean ± SD	Possible Range	Actual Range
Environmental characteristics	Health behavior		2.83 ± 0.46	1–4	1.67–3.90
	Social support		5.42 ± 1.07	1–7	2.25–7.00
Claudication pain	Total		1.20 ± 1.22	0–4	0–4
	No difficulty	51 (37.0)			
	Slightly difficulty	41 (29.7)			
	Some difficulty	19 (13.8)			
	Much difficulty	21 (15.2)			
	Unable to do	6 (4.3)			
Functional status	Walking distance		0.71 ± 0.34	0–1	0–1
	Walking speed		0.51 ± 0.28	0–1	0–1
	Stair climbing		0.71 ± 0.34	0–1	0–1
General health perception	Perceived health status		2.78 ± 0.79	1–5	1–5
	Perceived PAD severity		3.65 ± 1.09	1–5	1–5
HRQOL	EQ-5D-5L utility score		0.81 ± 0.17	−0.07–1.00	0.16–1.00
	EQ-5D-5L VAS		66.84 ± 18.99	0–100	0–100

Note: EQ-5D-5L, EuroQol-5 dimensions-5 levels; HRQOL, health-related quality of life; PAD, peripheral artery disease; SD, standard deviation; VAS, visual analog scale.

**Table 3:** EQ-5D-5L characteristics of participants (*n* = 138).

EQ-5D-5L Dimension	No Problems	Slight Problems	Moderate Problems	Severe Problems	Unable to/ Extreme Problems
Mobility	54 (39.1)	46 (33.3)	20 (14.5)	17 (12.3)	1 (0.7)
Self-care	117 (84.8)	17 (12.3)	2 (1.4)	1 (0.7)	1 (0.7)
Usual activities	86 (62.3)	36 (26.1)	8 (5.8)	6 (4.3)	2 (1.4)
Pain/discomfort	59 (42.8)	54 (39.1)	16 (11.6)	7 (5.1)	2 (1.4)
Anxiety/depression	96 (69.6)	38 (27.5)	2 (1.4)	1 (0.7)	1 (0.7)

Note: Data are expressed as the *n* (%).

**Table 4:** Multiple linear regression analysis in relation to HRQOL (*n* = 138).

Variables	Subcategories	β	SE	*p*
Duration of PAD		0.036	0.001	0.527
Using walking assistive device		0.036	0.036	0.590
Environmental characteristics	Health behavior	0.027	0.022	0.657
	Social support	−0.010	0.009	0.860
Claudication pain		−0.188	0.010	0.012
Functional status	Walking distance	0.371	0.049	<0.001
	Walking speed	0.001	0.055	0.999
	Stair climbing	0.315	0.047	0.001
General health perception	Perceived health status	0.091	0.014	0.176
	Perceived PAD severity	−0.007	0.009	0.911

Note: Adjusted for sex, age, and level of education. F = 15.92, *p* < 0.001, adjusted R^2^ = 0.604, Durbin − Watson = 1.952.

## Data Availability

The data are not publicly available in order to protect of subjects’ privacy and confidentiality. The data presented in this study are available on reasonable request from the corresponding author.
